# Supplementary figures and images for: Development of an Open-source Low-cost Pressure Myography and Cardiac Flow Simulator, HemoLens, for Mechanical Characterization of Native and Engineered Blood Vessels
Source: bioRxiv. 2025 May 4:2025.04.29.651300. Preprint. [Version 1] doi: 10.1101/2025.04.29.651300 (PMC12247934; doi:10.1101/2025.04.29.651300)

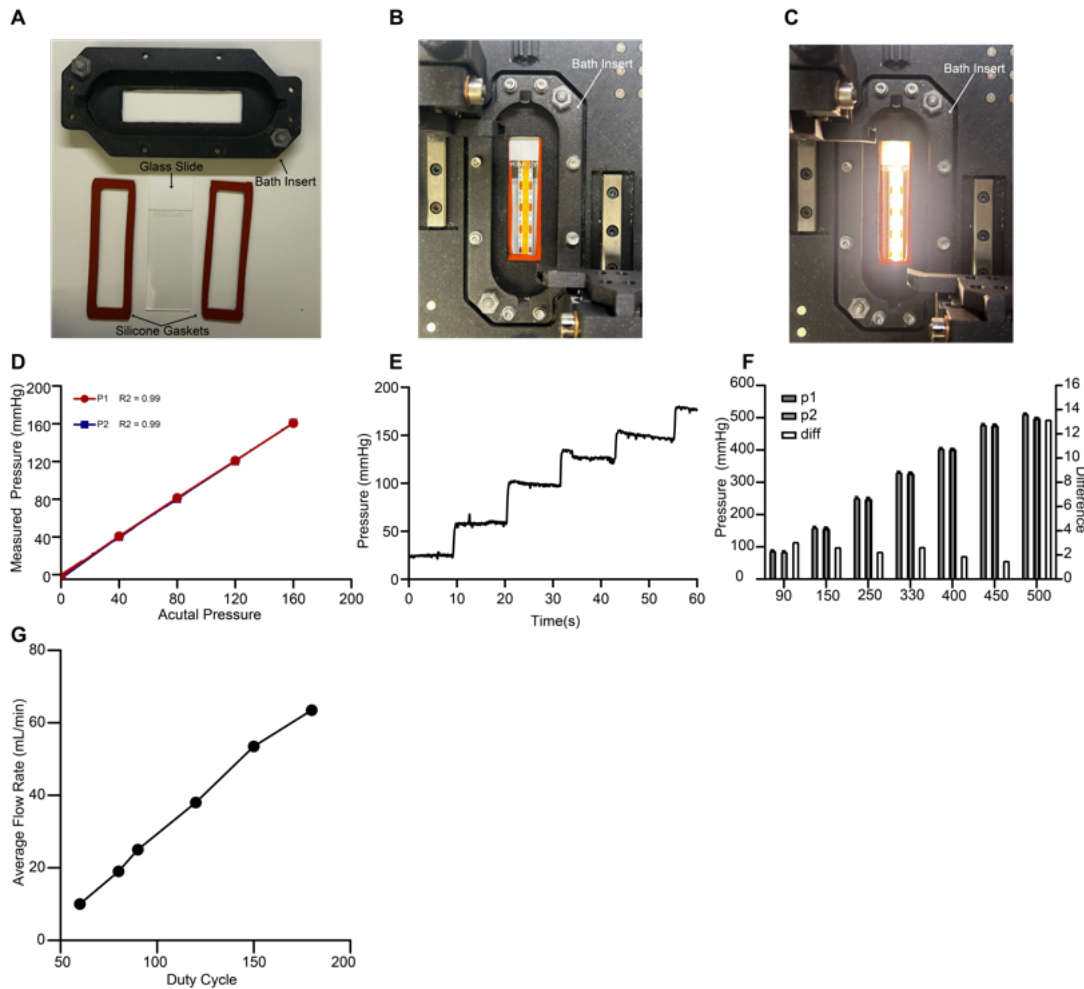

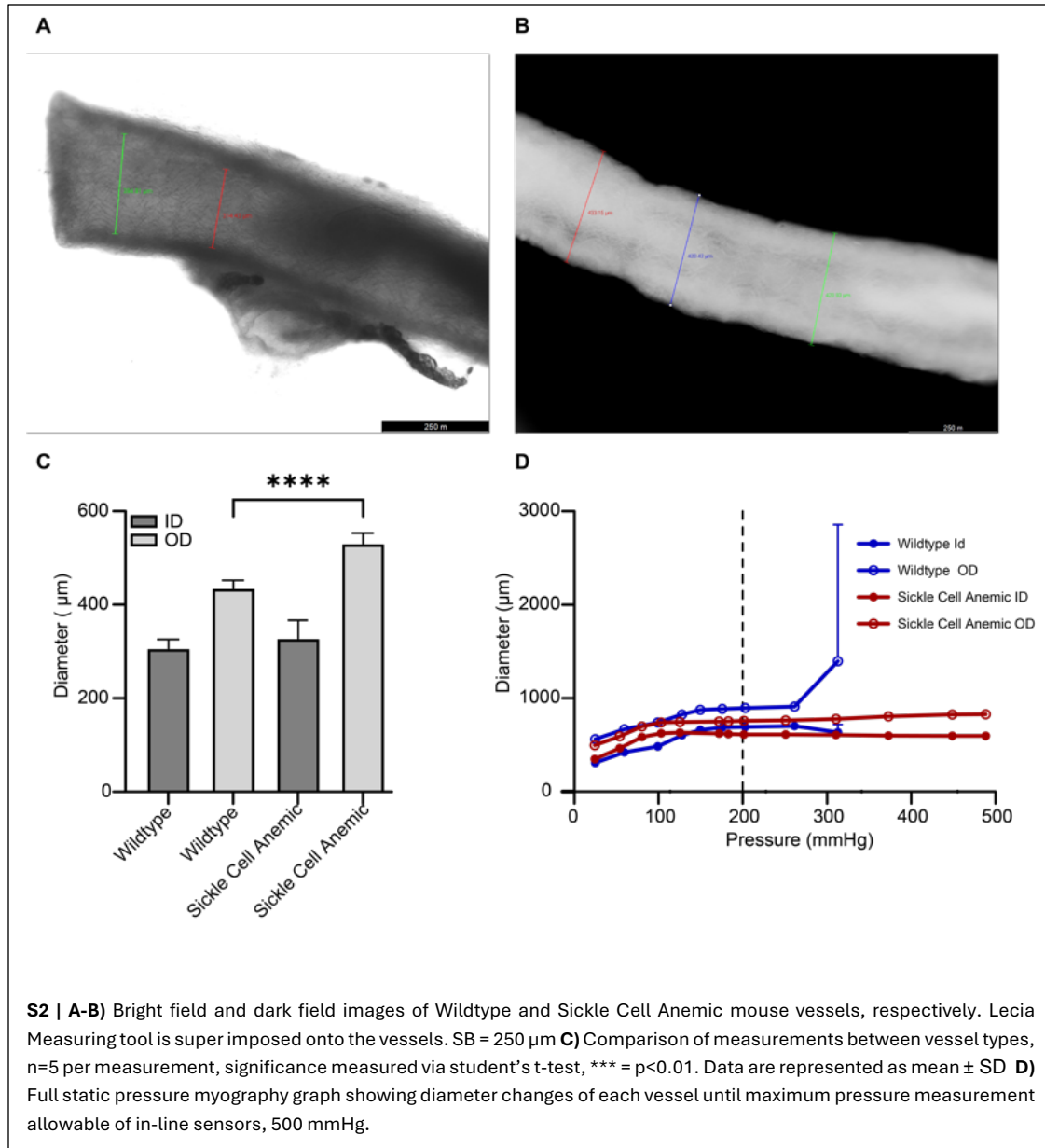

Supplement: Supplement 1 [file media-1.pdf]
